# Regulation profiles of e-cigarettes in the United States: a critical review with qualitative synthesis

**DOI:** 10.1186/s12916-015-0370-z

**Published:** 2015-06-03

**Authors:** Marie-Claude Tremblay, Pierre Pluye, Genevieve Gore, Vera Granikov, Kristian B. Filion, Mark J. Eisenberg

**Affiliations:** Department of Family Medicine, Faculty of Medicine, McGill University, 5858 Chemin de la Côte-des-Neiges, 3rd floor, Montreal, QC H3S 1Z1 Canada; Life Sciences Library, McGill University, 3655 Promenade Sir William Osler, Montreal, QC H3G 1Y6 Canada; Centre for Clinical Epidemiology, Lady Davis Institute, Jewish General Hospital, 3755 Côte-Ste-Catherine Road, Montreal, QC H3T 1E2 Canada; Department of Epidemiology, Biostatistics and Occupational Health, McGill University, 1020 Pine Avenue West, Montreal, QC H3A 1A2 Canada; Division of Clinical Epidemiology, Jewish General Hospital, 3755 Côte-Ste-Catherine Road, Montreal, QC H3T 1E2 Canada; Divisions of Cardiology and Clinical Epidemiology, Jewish General Hospital/McGill University, 3755 Côte-Ste-Catherine Road, Suite H-421.1, Montreal, QC H3T 1E2 Canada

**Keywords:** E-cigarette regulation, Public health policy, United States

## Abstract

**Background:**

Electronic cigarettes (e-cigarettes) have been steadily increasing in popularity since their introduction to US markets in 2007. Debates surrounding the proper regulatory mechanisms needed to mitigate potential harms associated with their use have focused on youth access, their potential for nicotine addiction, and the renormalization of a smoking culture. The objective of this study was to describe the enacted and planned regulations addressing this novel public health concern in the US.

**Methods:**

We searched LexisNexis Academic under Federal Regulations and Registers, as well as State Administrative Codes and Registers. This same database was also used to find information about planned regulations in secondary sources. The search was restricted to US documents produced between January 1^st^, 2004, and July 14^th^, 2014.

**Results:**

We found two planned regulations at the federal level, and 74 enacted and planned regulations in 44 states. We identified six state-based regulation types, including i) access, ii) usage, iii) marketing and advertisement, iv) packaging, v) taxation, and vi) licensure. These were further classified into 10 restriction subtypes: sales, sale to minors, use in indoor public places, use in limited venues, use by minors, licensure, marketing and advertising, packaging, and taxation. Most enacted restrictions aimed primarily to limit youth access, while few regulations enforced comprehensive restrictions on product use and availability.

**Conclusions:**

Current regulations targeting e-cigarettes in the US are varied in nature and scope. There is greater consensus surrounding youth protection (access by minors and/or use by minors, and/or use in limited venues), with little consensus on multi-level regulations, including comprehensive use bans in public spaces.

**Electronic supplementary material:**

The online version of this article (doi:10.1186/s12916-015-0370-z) contains supplementary material, which is available to authorized users.

## Background

Electronic cigarettes (e-cigarettes) are battery-powered devices that vaporize a flavored propylene glycol or glycerin solution, with or without nicotine, to simulate cigarette smoking. Since their introduction to North American markets in 2007, studies have shown increased awareness and use of e-cigarettes, both among high school students and young adults. The e-cigarette global industry is projected to reach US $10 billion by 2017 [1]. Although e-cigarettes have the potential to act as harm reduction devices due to the absence of combustion-related toxins and carcinogens produced by conventional cigarettes, the long-term health effects of vapor inhalation are unknown. Other public health concerns include e-cigarettes’ potential for nicotine addiction in youth, the renormalization of a smoking culture, and accidental nicotine poisoning among children [2–4]. Despite these concerns, e-cigarettes have largely evaded regulation given the ambiguity surrounding their classification as tobacco products, consumer products, or medical devices. Our objective was to conduct a critical review of current and planned legislation targeting e-cigarettes at the US federal and state levels, in the aim of describing the different regulatory approaches that will inform the future availability of and access to e-cigarettes.

## Methods

### Search strategy

This critical review was conducted following a pre-specified protocol and is reported according to the MOOSE (Meta-analysis Of Observational Studies in Epidemiology) guidelines [5], with the literature search described using a PRISMA (Preferred Reporting Items for Systematic Reviews and Meta-Analyses) flow diagram [6]. With guidance from a Law librarian, two specialized health librarians developed the search strategy and conducted the search in July 2014. The search was conducted in the subscription-based legal databases available in LexisNexis Academic, under Federal Regulations and Registers as well as State Administrative Codes and Registers, using the keywords “electronic cigarette*” OR “e-cigarette*”. Secondary sources, including US Law reviews, journals, as well as newspaper articles, were also searched using LexisNexis Academic, using the keywords “electronic cigarette*” OR “e-cigarette*”. The search was restricted to documents produced in the US between January 1^st^, 2004, and July 14^th^, 2014. In addition, six specialized websites were used to supplement and validate the search [7–12].

### Study selection

We searched for regulations targeting e-cigarettes at the US federal or state level, specifically enacted regulations and laws (hereafter collectively referred to as “regulations”), as well as future regulations proposed as of July 1^st^, 2014. For the purposes of this review, an enacted regulation was considered an effective regulation or law (act, statute, code) or an enacted bill (signed into law), while a planned regulation was deemed a regulation or law draft presented to legislature for discussion, and mentioned in a bill or in proposed rules by a specific agency. Regulatory documents were included if they were i) issued at the US federal or state level and ii) explicitly targeted e-cigarettes, electronic smoking devices, electronic nicotine delivery devices, or vapor products. Documents concerning municipal and county regulations were excluded. In addition, documents addressing only nicotine-containing or tobacco-derived products were excluded, unless they explicitly included e-cigarettes as one of these products.

### Data extraction and qualitative synthesis

Two reviewers performed data extraction and traditional data-near qualitative content analysis [13]. For each included regulation, we extracted the following characteristics: i) level of regulation (federal or state); ii) status of regulation (enacted regulation; proposed regulation [bill] just signed into law; planned regulation); iii) year of introduction to legislature or of enactment; iv) description of regulation; and v) legal citation of act, statute, rule, or bill.

From regulation descriptions, similar groupings were identified using existing regulation typologies [11, 14–16]. Data were tabulated by level (federal, state) and by state for side-by-side comparison and compiled by regulation types. Regulation profiles were identified in an inductive interpretive manner by the first author and validated by the second author. Regulation profiles were defined as specific combinations of regulation types that illustrate the regulatory approach of a particular state.

## Results

**Fig. 1:**
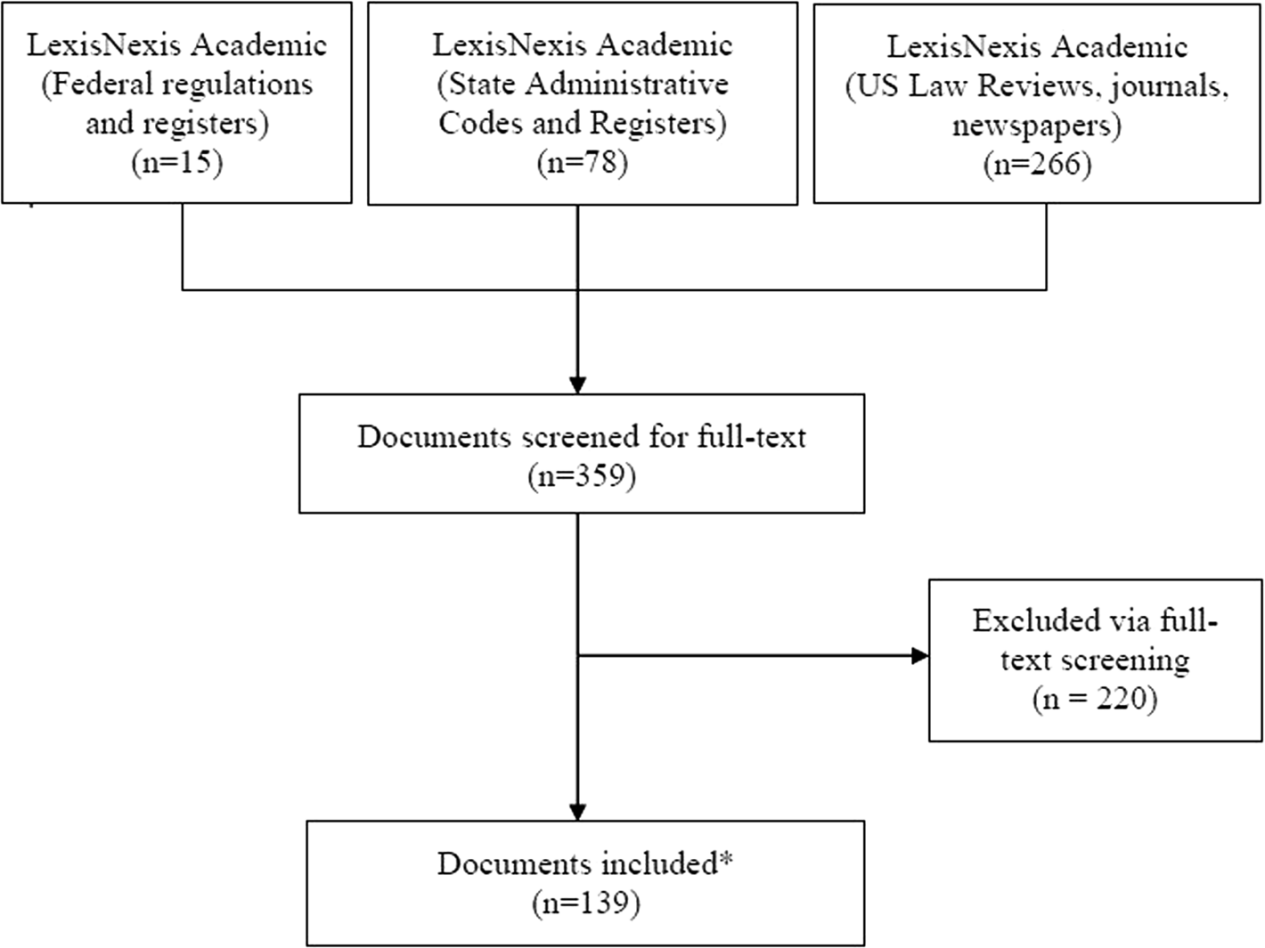
PRISMA flow diagram of systematic review literature search. * The 139 documents discussed 75 enacted or planned regulations, including two at the federal level and 73 at the state level (including the District of Columbia). US, United States

Our systematic search yielded 359 potentially relevant documents (Fig. 1). Searches in primary sources produced 15 federal records and 78 state records, while searches in secondary sources resulted in 266 records. Following full-text screening, 139 documents met our inclusion criteria, constituting two planned federal regulations and 74 enacted and planned regulations in 44 states.

### Federal regulation of e-cigarettes

In 2009, the Food and Drug Administration (FDA) of the Department of Health and Human Services attempted to regulate e-cigarettes as drug-delivery devices under the Federal Food, Drug and Cosmetic Act [17]. In a judgement dated December 2010, the federal appeals court ruled that the FDA could not regulate e-cigarettes as such, unless the product was marketed as a smoking cessation tool or a therapeutic device [11]. Instead, the court ruled that e-cigarettes would be considered as tobacco products under the Family Smoking Prevention and Tobacco Control Act, which allows the FDA to oversee products derived from tobacco, including nicotine [18].

Although the FDA stated its intent to regulate e-cigarettes as tobacco products in 2011, our search did not produce any federally enacted regulations of e-cigarettes. However, we found records of two proposed federal regulations relevant to e-cigarettes (Additional file 1). The first was advanced by the Department of Transportation on September 15^th^, 2011 [19]. This ruling aimed to ban the use of e-cigarettes on all passenger aircrafts flying to or from the US [19]. Although the final ruling was to be issued in September 2014, it was still pending as of November 2014. The second was proposed by the FDA on April 25^th^, 2014, aiming to subject e-cigarettes and other tobacco products to the FDA’s authority under the Federal Food, Drug, and Cosmetic Act, as amended by the Family Smoking Prevention and Tobacco Control Act [20]. These proposed regulations would subject e-cigarettes to the same requirements as conventional cigarettes and tobacco products. They would also prohibit the sale of such products to individuals under the age of 18 years, in addition to requiring the display of health warnings on these products’ packages and advertisements [20]. These rules imply that manufacturers have to disclose their products’ ingredients on their packaging and ban the sale of these products in vending machines as well as the distribution of free samples. The FDA is due to issue its final ruling in June 2015.

### State regulations of e-cigarettes

**Fig. 2:**
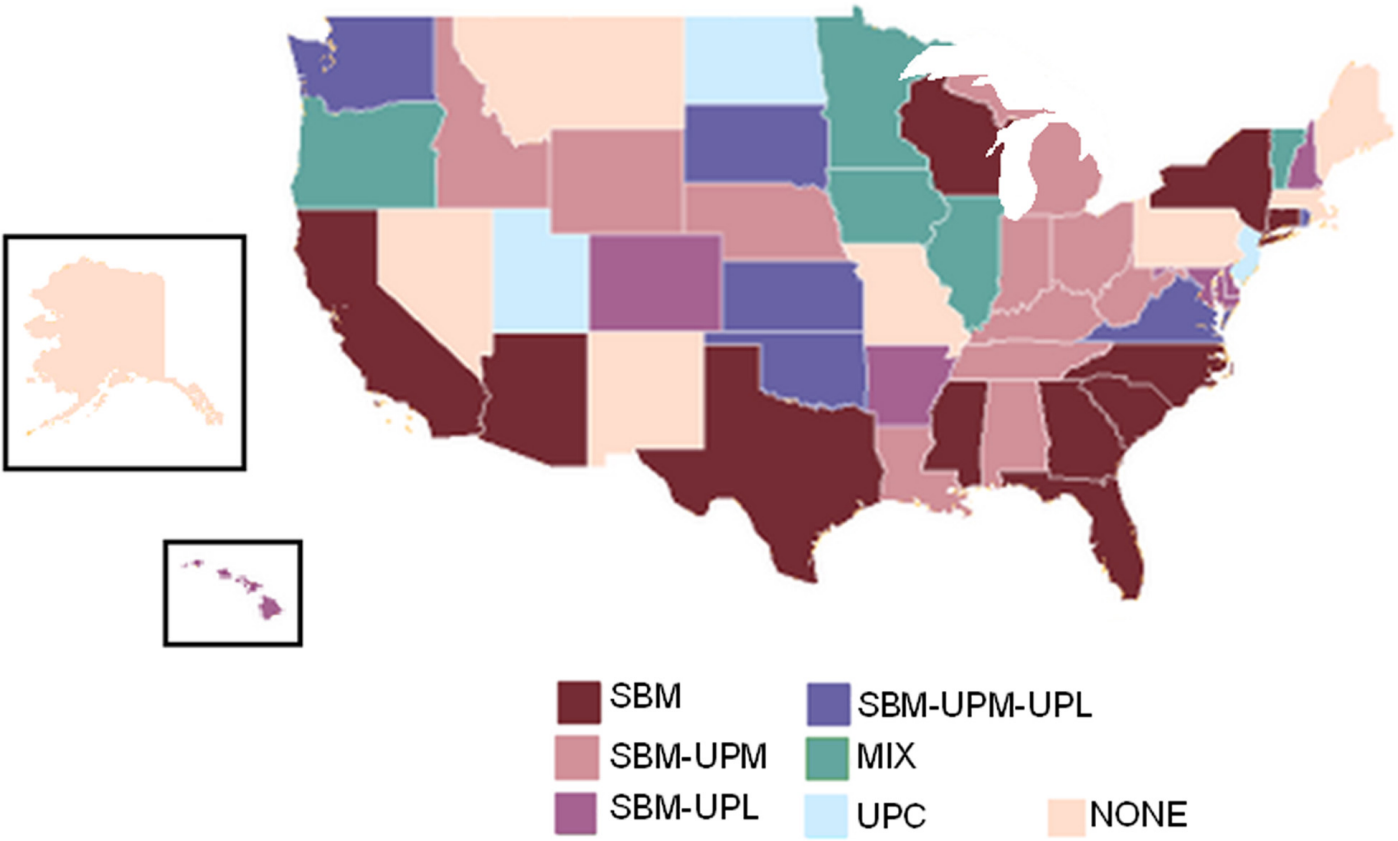
State-by-state comparison of e-cigarette regulation profiles. Mix, Mixed regulations; SBM, Sale to minors ban; SBM-UPL, Sale to minors ban and prohibited use in limited venues; SBM-UPM, Sale to minors ban and prohibited use of e-cigarette by minors; SBM-UPL-UPM, Sale to minors ban, prohibited use by minors, and in limited venues; UPC, Use prohibited comprehensively in indoor public places. Map created with [24]

In the absence of enacted federal regulation, US states have been very proactive in regulating e-cigarettes. As of July 2014, 44 states had planned or enacted 74 regulations addressing e-cigarettes, electronic smoking devices, or vapor products (Additional file 2). Overall, six types of state regulations were identified, including i) access, ii) usage, iii) marketing and advertisement, iv) packaging, v) taxation, and vi) licensure (Table 1). These regulation types were further classified into 10 subtypes: sale ban, sale to minors ban, use prohibited comprehensively in indoor public places, use prohibited in limited venues, use by minors prohibited, licensure restrictions, marketing and advertising restrictions, marketing and advertising to minors restrictions, packaging requirements, and taxation. We found that certain regulation subtypes, including the sale to minor ban (n = 38), use by minors prohibited (n = 18), and use prohibited in limited venues (n = 16), were enacted most frequently, whereas use prohibited comprehensively (n = 3), packaging requirements (n = 3), as well as regulations addressing taxation (n = 2), licensure (n = 2), and marketing or advertisement (n = 1) were relatively infrequent (Fig. 2). All-inclusive sale bans (n = 1), such as Oregon’s, were also uncommon.

**Table 1:** Description of e-cigarette regulation types and sub-types

Regulation type	Regulation sub-type	Description
Access	SB	Unilateral sale ban of e-cigarettes
	SBM	Sale ban of e-cigarettes to minors (typically under 18 years)
Use	UPC	Use of e-cigarettes prohibited in all smoke-free public places (i.e., non-hospitality workplaces, restaurants, bars, and gambling facilities), often in accordance with local smoke-free laws
	UPL	Use of e-cigarettes prohibited in certain specific venues (i.e., school and public education facilities, child care facilities, state workplaces, or department of corrections property)
	UPM	Possession and use of e-cigarettes by minors prohibited (typically under 18 years)
Licensure	L	Required licensure for retailers to facilitate sales tracking
Marketing and advertising	MA	Constraints imposed on marketing and advertisement of e-cigarettes, including television advertisement restrictions, or requirements that e-cigarettes be stored for sale behind a counter
	MAM	Prohibition against all forms of marketing or advertisement of e-cigarettes to minors
Packaging	P	Requirement that e-cigarette packages be childproof or conform to certain standards, including the display of health warnings or listing of product ingredients
Taxation	T	Taxes on e-cigarettes and/or e-cigarette liquid, often by virtue of classifying e-cigarettes as a tobacco product, rendering it subject to local tobacco taxes

L, Licensure; MA, Marketing and advertising to minors; MAM, Marketing and advertising to minors; P, Packaging; SB, Sale ban; SBM, Sale to minors ban; T, Taxation; UPC, Use prohibited comprehensively; UPL, Use prohibited in limited venues; UPM, Use prohibited by minors

Regulation profiles, which are a specific combination of regulation types, are presented in Fig. 2. While the clustering of similar regulatory profiles across eastern and southern states suggests the possibility of geographic or political influence, specific analyses remain outside the scope of this review. At present, the majority of states (n = 35) have implemented limited and targeted regulations (i.e., sale to and/or use by minors, use in limited venues), whereas a minority (n = 8) have enacted relatively comprehensive regulations (i.e., use in public places, mixed varied regulations). A total of seven states had no regulations targeting e-cigarettes.

## Discussion

Our study was designed to describe US federal and state-level regulation profiles of e-cigarettes, with a view to inform the future of e-cigarette availability and use. Overall, we found a large dataset of regulations targeting e-cigarettes, both proposed and enacted [15]. The available evidence suggests that state-level regulations are varied in their approach and scope, while federal regulations remain currently absent. However, the proposed federal regulations extending the FDA’s authority over e-cigarettes, if enacted, would serve to provide greater consistency in the policy approaches targeting this novel public health concern. With the implementation of these proposed federal rules, the US would join at least 22 other countries currently regulating e-cigarettes as tobacco products (as opposed to consumer products or therapeutic devices) and at least 29 other countries prohibiting sales to minors [21].

A key observation from our results is the principal focus on youth protection, rather than broad public health concerns. Use ban in limited venues (often in schools and childcare facilities), as well as regulations that limit marketing and advertisement to minors also primarily aim to reduce access to youth. Of note, however, is the absence of any current or planned regulations targeting flavored e-cigarette liquid (or “e-juice”). Recent evidence suggests that interest in e-cigarette flavoring is low among adolescents who do not smoke traditional or e-cigarettes [21]. However, these data remain difficult to interpret given the absence of comparator groups of smoking and non-smoking adolescents who use e-cigarettes, for whom flavorings may significantly contribute to product interest. Additional research will be required to conclusively establish the importance of flavorings on e-cigarette uptake among smoking and non-smoking youths. Nevertheless, following the release of the FDA’s proposed rules in April 2014, a letter signed by 29 attorneys general called for tougher regulations for children, including a ban on flavorings other than tobacco and menthol, advertising restrictions, and a ban on youth-targeted marketing, similar to those enforced for conventional cigarettes [22].

A useful lens through which to consider how to mitigate the potential harms associated with e-cigarette use is Geoffrey Rose’s model of high-risk versus population prevention [23]. High-risk strategies target groups for whom intervention offers the greatest benefit by reducing their exposure to a possible cause of harm [23]. For instance, minors may constitute a high-risk group that is more vulnerable to nicotine addiction relative to adults. Regulations that limit youth exposure to the product could therefore restrict minors’ access to and use of e-cigarettes. Typically, high-risk strategies are relatively politically palatable as they avoid impinging upon the freedoms of those deemed to be at a lower risk. Such public favor likely accounts for the frequency of youth-targeted interventions across states. However, an important limitation of high-risk strategies is their failure to address the social determinants that encourage behaviors such as nicotine consumption, or vaping [23].

An alternative to a high-risk strategy is a population approach to prevention, which aims to minimize the barriers preventing people from making healthier choices [23]. This comparatively radical strategy is typically enforced through comprehensive multi-level regulations, including bans on product sales or use. Population prevention targets social norms in the aim of modifying the acceptability of a potentially harmful product in society. Because this approach often takes the form of broad-spanning legislation undermining personal freedoms, population prevention may fall into disfavor for its perceived paternalism. Accordingly, few states have implemented population prevention strategies aimed at restricting the public availability and use of e-cigarettes.

Ultimately, e-cigarette regulations should be devised on the merits of their suitability and feasibility, taking into account the existing regulatory framework in a given state or country. A recent report drafted by the WHO outlined some primary objectives governments should bear in mind when drafting regulation for e-cigarettes [21]. These include restricting e-cigarette uptake by vulnerable groups or non-smokers, and minimizing potential health risks to users and non-users. To this effect, the WHO recommends that countries consider prohibiting unproven health claims about e-cigarettes, banning the use of e-cigarettes in indoor public places, restricting e-cigarette advertising, promotion and sponsorship, standardizing product design, enforcing the display of health warnings on packaging, as well as prohibiting sale to minors [21]. As the proposed US federal regulations only touch upon some of these concerns, states will likely continue to implement complementary regulations to address potential shortcomings.

In comparison to state laws that govern conventional cigarettes, those overseeing e-cigarettes are more variable as there are currently no federal regulations in the likes of those governing tobacco products, such as the Family Smoking Prevention and Tobacco Control Act. This federal rule prohibits the sale of conventional cigarettes and other tobacco products to minors, in addition to imposing constraints on tobacco products’ packaging, marketing, advertisement, and sponsorship. While state regulations of conventional and e-cigarettes thus present important differences, in both cases, individual states remain accountable to implement and amend smoke-free air laws, prohibit tobacco and e-cigarette use in specific venues, and increase excise taxes on these products.

Previous reviews of e-cigarette regulations have presented limited and partial portraits of the regulatory system put in place in the US, focusing mostly on e-cigarette indoor use and youth access laws [5, 15]. In contrast, this review presents a comprehensive overview of the federal and state-level regulatory approaches targeting e-cigarettes, including planned and enforced regulations addressing usage and access, but also marketing and advertisement, packaging, and taxation.

### Limitations

Our results should be interpreted in the context of several potential study limitations. First, all information presented herein is subject to availability in LexisNexis Academic, between January 1^st^, 2004, and July 14^th^, 2014. Second, although additional websites [7–12] were used to complement our search strategy, certain non-codified or planned regulations may have been missed due to their absence within databases. Third, municipal regulations were excluded as these were beyond the scope of our research. Finally, documents limited to nicotine-containing or tobacco-derived products were excluded, unless these explicitly included e-cigarettes.

## Conclusions

Overall, highly targeted regulation profiles, such as those aimed at youth protection, are popular in the US, while radical, multi-targeted regulation profiles remain relatively unusual. Differences in states’ approaches to regulation may be due to the ease of implementing youth-specific restrictions, as compared to regulations aiming to restrict product use for all consumers. Given the lack of data concerning their safety as consumer products, and their potential efficacy as smoking cessation devices, it is unclear to what extent and by what means e-cigarettes should be regulated. In the meantime, regulations should remain highly adaptable in order to respond to any emerging evidence concerning this new product’s potential harms and benefits.

## Additional files

Additional file 1:
**Enacted and proposed US federal and state regulations of e-cigarettes.**
Additional file 2:
**E-cigarette regulation profiles of 43 states.**

## Abbreviations

E-cigarettes: Electronic cigarettes
FDA: U.S. Food and Drug Administration
US: United States
