# Supplementary material for: Regulation profiles of e-cigarettes in the United States: a critical review with qualitative synthesis
Source: BMC Med. 2015 Jun 3;13:130. doi: 10.1186/s12916-015-0370-z (PMC4480885; doi:10.1186/s12916-015-0370-z)
Supplement: Additional file 1: — Enacted and proposed US federal and state regulations of e-cigarettes. [file 12916_2015_370_MOESM1_ESM.docx]

Additional file 1. Enacted and proposed US federal and state regulations of e-cigarettes.

| **Level** | **Status** | **Year*** | **Regulation Types†** | **Legal Citations of Statutes, Acts, Rules, and Bills** |
| --- | --- | --- | --- | --- |
| **Federal: DHHS, FDA** | P | 2011 | UPS (in aircrafts on all domestic and international flights to or from the US)  . | 49 USC §41706, 41702; 14 CFR Part 252; Docket No. DOT-OST-2011-0044 |
| **Federal: DOT** | P | 2014 | SBM  MA  P | 21 CFR Parts 1100, 1140, 1143; Docket No. FDA-2014-N-0189 |
| **AL** | E | 2013 | SBM-UPM | Ala. Code §28-11-2 |
| **AZ** | E | 2010 | SBM | Ariz. Rev. Stat. Ann. §13-3622 |
| **AR** | E | 2013 | SBM | Ark. Stat. Ann. §5-27-233 |
|  | E | 2013 | UPL (school district property) | Ark. Code. Ann. §§4-16-101, 6-21-609 |
| **CA** | E | 2011 | SBM | Cal. Code §119405 |
|  | P | 2013 | UPL (public buildings, schools, and day care center)  MAM (restrict television advertisements and prohibit marketing to minors) | Cal. SB 648 |
| **CO** | E | 2011 | SBM | Colo. Rev. Stat. §§18-13-121, 24-35-501 et seq., 25-14-30, 22-32-109, 25-14-204 |
|  | E | 2011 | UPL (school property, unless approved by FDA as cessation devices) | Colo. Code Regs. §2509-8 |
| **CT** | S | 2014 | SBM (Effective 10/01/2014) | Conn. SB 24, Pub. Act No. 14-76 |
| **DC** | E | 2013 | UPL (public libraries) | 19-810 D.C. Code Mun. Regs §810.5 |
|  | E | 2013 | UPL (playgrounds, public recreational facilities and bus stops, in parity with the District of Columbia Smoking Restriction Act of 1979) | D.C. Law 3-22; D.C. Code Ann. §§7-1701 et seq. |
| **DE** | E | 2013 | UPL (state workplace property, indoor and outdoor) | State of Delaware Tobacco-Free Workplace Policy |
|  | S | 2014 | SBM (signed by governor 06/12/2014) | Del. HB 241 |
|  | P | 2014 | UPC (in parity with Delaware’s 2002 Clean Indoor Air Act) | Del. HB 309 |
| **FL** | E | 2014 | SBM | Fl. Rev. Stat. §569.14, §322.056; Fl. Stat. §877.112 |
| **GA** | E | 2014 | SBM | Georgia HB 251, Georgia §16-12-171.1 |
| **HI** | E | 2013 | SBM | Haw. Rev. Stat. §709-908 |
|  | E | 2014 | UPL (Department of Health property) | Haw. Department of Health, Intra-Departmental Directives, no. 13-03.01 et seq. |
|  | P | 2013 | UPC (in parity with Hawaii’s anti-smoking statute, including workplaces and public places) | Haw. SB 2495 |
|  | P | 2013 | MAM (requires that e-cigarettes be stored for sale behind a counter, advertisements to be placed four feet off the floor) | Haw. SB 652 |
|  | P | 2014 | L | Haw. SB 2495 |
| **ID** | E | 2012 | SBM-UPM | Idaho Code Ann. §39-5702 et seq. |
| **IL** | E | 2013 | SBM | Ill. Stat. Ann 720 ILCS 675/1.5 |
|  | S | 2015 | P (effective 01/01/2015) | Ill. HB 5689, III. Pub. Act 098-1021 |
| **IN** | E | 2013 | SBM-UPM | Ind. Code §§35-46-1-1.5, 35-46-1-10, 35-46-1-10.2, 7.1-3-18.5-8 |
|  | P | 2014 | T (OTP rate of 24% of wholesale) | Ind. HB 1174 |
| **IA** | S | 2014 | SBM (signed by governor 05/23/2014, effective 07/01/2014) | Iowa HB 2109 |
|  | S | 2014 | L (signed by governor 05/23/2014, effective 07/01/2014) | Iowa HB 2109 |
| **KS** | E | 2012 | SBM-UPM | Kan. Stat. Ann. §§79-3301, 3303, 3321 |
|  | E | 2010 | UPL (Department of Corrections property and grounds, by both employees and inmates) | Kan. Admin. Regs. §123-2-111 |
| **KY** | S | 2014 | SBM-UPM (signed by governor 04/10/2014) | Ken. SB 109, Ken. Rev. Stat. §§438.30-438.340, 438.350 |
|  | P | 2014 | T (defines e-cigarettes as tobacco products, 15% OTP tax) | Ken. HB 319 |
| **LA** | S | 2014 | SBM-UPM (signed by governor 05/29/2014) | Lou. SB 12, Lou. Rev. Stat. §§14 :91.8, 26 :910, 910.1(A), 14 :91.6(B)(6)(7), 26 :901(28) |
| **MD** | E | 2011 | UPL (MARC commuter rail system trains) | Md. Code Regs. 13A.02.04.01-.07 |
|  | E | 2012 | SBM | Md. Code Ann. Health-Gen. §24-305 |
| **MA** | P | 2013 | UPL (public school grounds, public areas and workplaces)  SBM | Mass. HB 3726 |
| **MI** | S | 2013 | SBM-UPM | Mich. HB 4997 |
| **MN** | E | 2010 | SBM | Minn. Stat. §609.6855 |
|  | E | 2013 | T (95% of the wholesale cost of any product containing or derived from tobacco) | Minn. Stat. §297F.01, subd. 19 |
|  | S | 2014 | UPL (agency building, state colleges and universities, day care facilities, health care facilities and clinics) (signed by governor 05/21/2014)  MA (bans retail sales of the products from mail kiosks, requires them to be kept behind store counter)  P | Minn. HB 2402 |
| **MS** | E | 2013 | SBM | Miss. Code Ann. §97-32-51 |
| **NE** | S | 2014 | SBM-UPM (effective 04/09/2014) | Neb. LB 863 |
| **NH** | E | 2010 | SBM  UPL (public educational facilities) | N.H. Rev. Stat. Ann. §126-K:2 et seq. |
| **NJ** | E | 2010 | SBM | N.J. Stat. Ann. §2A :170-51.4 |
|  | E | 2010 | UPC (workplaces and indoor public areas, in parity with New Jersey’s Smoke-Free Air Act of 2006) | N.J. Stat Ann §§26-3D-55 et seq., 2C :33-13.1, 26 :3A2-20.1, N.J. Admin. Code §§10 :128-4.6, 10 :122C-7.2 |
| **NY** | E | 2011 | SBM | N.Y. Code §1399-aa, §1399-cc |
|  | P | 2013 | UPC (public areas including bars, restaurants, offices, and other public indoor spaces, in parity with New York State’s Clean Indoor Air Act) | N.Y. AB 8178 |
|  | P | 2014 | T (classifies e-cigarettes as tobacco products; 75% excise tax on e-cigarettes) | N.Y. AB 8594 |
|  | P | 2014 | UPL (certain indoor areas) | N.Y. AB 10182 |
| **NC** | E | 2013 | SBM | N.C. Gen. Stat. Ann. §14-313 |
|  | S | 2014 | T (5 cents per milliliter tax on e-cigarette liquid) (signed by governor on 05/29/2014) | N.C. HB 1050 |
| **ND** | E | 2012 | UPC (public areas, including non-hospitality workplaces, restaurants, bars, and gambling facilities, in accordance with North Dakota’s smoke-free law) | N.D. Cent. Code §23-12-09 |
| **OH** | S | 2014 | SBM-UPM (signed by governor 03/04/2014, effective 08/02/2014) | Ohio HB 144, Ohio Rev. Code §§2151.87, 2927.02, 2927.021, and 2927.022 |
| **OK** | E | 2014 | UPL (state properties, including Department of Corrections facilities, vehicles, and grounds) | Okla. Exec. Order No. 2013-43, 31, Okla. Reg. 340 |
|  | S | 2014 | SBM-UPM (Effective 11/01/2014) | Okla. SB 1602 |
| **OR** | E | 2009 | SB (Department of Justice settlement prohibits sale of e-cigarettes until they are FDA approved, or until court rules FDA cannot regulate them and scientific research can prove them safe) | Or. Judicial order |
|  | E | 2013 | UPL (State agency buildings and grounds, university-owned or controlled properties) | Or. Admin. R. 571-050-0005, 576-040-0010—0015, 576-015-0020; Exec. Order No. 12-13, 51 No.9 Or. Bull. 4 |
| **RI** | S | 2014 | SBM-UPM (signed by governor 06/30/2014, effective 07/01/2014)  L (signed by governor 06/30/2014, effective 01/01/2014) | R.I. HB 7021, R.I. Rev. Stat. §§11-9-13 et seq., 11-9-14, Chap. 11-9 |
| **SC** | E | 2013 | SBM | S.C. Code Ann. §§16-17-500 et seq. |
| **SD** | E | 2014 | SBM-UPM | S.D. §§34-46-2 et seq. Chap 34-46 |
|  | E | 2014 | UPL (Department of Corrections facilities and on grounds thereof, by both employees and inmates) | S.D. Department of Corrections Policy, 1.3.C.7 – Tobacco Products and Electronic Cigarettes – Use and Possession |
| **TN** | E | 2011 | SBM-UPM | Tenn. Code Ann. §39-17-1501 et seq. |
| **TX** | E | 2013 | UPL (to be eligible to receive CPRIT funding, a CPRIT-funded entity shall certify that the entity has adopted and enforces Tobacco-Free Workplace policy, including e-cigarettes) | Tex. Admin. Code §703.20 |
| **UT** | E | 2010 | SBM-UPM | Ut. Code Ann. §§77-39-101, 76-8-311.3, 76-10-101 et seq, 53A-11-908, 26-38-1 et seq., 58-37-8, 10-8-41.6, 17-50-333, 41-6a-1717 |
|  | E | 2012 | UPC (non-hospitality workplaces, restaurants, bars, and gambling facilities, in parity with Utah Indoor Clean Air Act) | Ut. Admin. Code r. 392-510-2—17 |
| **VT** | E | 2012 | SBM-UPM | Vt. Stat. Ann. Tit. 7, §1001 et seq. |
|  | E | 2014 | UPL (school grounds and at child care facilities, both indoors and outdoors) | Vt. Act No. 135 (H. 217) |
|  | E | 2015 | P (effective 01/01/2015) | Vt. Act No. 0188 |
| **VA** | E | 2014 | SBM-UPM | Vir. §18.2-371.2 Chapter 357 |
|  | E | 2014 | UPL (school property) | Vir. §22.1-79.5 – Chapter 326 |
| **WA** | E | 2013 | SBM-UPM | Wash. Rev. Code §26.28.080 |
|  | E | 2014 | UPL (community colleges and universities) | Wash. Admin. Code §§132Q-30-231, 132L-136-010, 132^E^-120-410, 172-122-310 |
| **WV** | E | 2014 | SBM-UPM | W.V. Code Ann. §16-9A-2 |
| **WI** | E | 2012 | SBM | Wis. Stat. Ann. §134.66 |
| **WY** | E | 2013 | SBM-UPM | Wyo. Stat. §14-3-301(a)(i) |

* In the case of planned regulation, year of introduction to legislature. In the case of an enacted regulation or signed regulation, year of enactment.

† Regulations explicitly addressing e-cigarettes, electronic smoking devices, electronic nicotine delivery devices and vapor products (not nicotine containing or tobacco-derived products, unless explicitly including e-cigarettes in these products).

Abbreviations: E = enacted regulation, P = planned regulation (bill or proposed rule), S = regulation (bill) signed into law, but not yet codified, UPC = use prohibited comprehensively in indoor public places, SBM = Sale to minors ban, UPL = use prohibited in limited venues, UPM = use by minors prohibited.

State Abbreviations: AZ = Arizona, AL = Alabama, AR = Arkansas, CA = California, CO = Colorado, CT = Connecticut, DC = District of Columbia, DE = Delaware, FL = Florida, GA = Georgia, HI = Hawaii, ID = Idaho, IL = Illinois, IA = Iowa, IN = Indiana, KS = Kansas, KY = Kentucky, LA = Louisiana, MA = Massachusetts, MD = Maryland, MI = Michigan, MN = Minnesota, MS = Mississippi, NE = Nebraska, NH = New Hampshire, NJ = New Jersey, New Mexico, NY = New York, NC = North Carolina, ND = North Dakota, OH = Ohio, OK = Oklahoma, OR = Oregon, Pennsylvania, RI = Rhode Island, SC = South Carolina, SD = South Dakota, TN = Tennessee, TX = Texas, UT = Utah, VT = Vermont, VA = Virginia, WA = Washington, WV = West Virginia, WI = Wisconsin, WY = Wyoming.
