# Supplementary material for: Regulation profiles of e-cigarettes in the United States: a critical review with qualitative synthesis
Source: BMC Med. 2015 Jun 3;13:130. doi: 10.1186/s12916-015-0370-z (PMC4480885; doi:10.1186/s12916-015-0370-z)
Supplement: Additional file 2: — E-cigarette regulation profiles of 43 states. [file 12916_2015_370_MOESM2_ESM.docx]

Additional file 2. E-cigarette regulation profiles of 43 states*

|  | **Profiles** | **States** |
| --- | --- | --- |
| 1 | SBM: States enforcing only a sale to minors ban (n = 11) | AZ, CA, CT, FL, GA, MS, NY, NC, SC, TX, WI |
| 2 | SBM, UPM: States enforcing a sale to minors ban and prohibiting use of e-cigarette by minors (n = 11) | AL, ID, IN, KY, LA, MI, NE, OH, TN, WV, WY |
| 3 | SBM, UPL: States enforcing a sale to minors ban and prohibiting use in limited venues (n = 7) | AR, CO, DC†, DE, HI, MD, NH |
| 4 | SBM, UPM, UPL: States enforcing a sale to minors ban, prohibiting use by minors and in limited venues (n = 6) | KS, OK, RI, SD, VA, WA |
| 5 | UPC: States prohibiting use in indoor public spaces (i.e., workplaces, restaurants, bars) (n = 3) | NJ, ND, UT |
| 6 | States using varied mix of regulations (n = 5) | IL, IA, MN, OR, VT |
|  | **Total** | 43 states with enacted or signed regulations |

*Includes only states with enacted and signed regulations. MA has thus been excluded.

†DC only prohibits e-cigarette use in limited venues.

Abbreviations: SBM = Sale to minors ban, UPC = use prohibited comprehensively in indoor public places, UPL = use prohibited in limited venues, UPM = use by minors prohibited.

State Abbreviations: AZ = Arizona, AL = Alabama, AR = Arkansas, CA = California, CO = Colorado, CT = Connecticut, DC = District of Columbia, DE = Delaware, FL = Florida, GA = Georgia, HI = Hawaii, ID = Idaho, IL = Illinois, IA = Iowa, IN = Indiana, KS = Kansas, KY = Kentucky, LA = Louisiana, MD = Maryland, MI = Michigan, MN = Minnesota, MS = Mississippi, NE = Nebraska, NH = New Hampshire, NJ = New Jersey, New Mexico, NY = New York, NC = North Carolina, ND = North Dakota, OH = Ohio, OK = Oklahoma, OR = Oregon, Pennsylvania, RI = Rhode Island, SC = South Carolina, SD = South Dakota, TN = Tennessee, TX = Texas, UT = Utah, VT = Vermont, VA = Virginia, WA = Washington, WV = West Virginia, WI = Wisconsin, WY = Wyoming.
